# Cyclopentenone Prostaglandins: Biologically Active Lipid Mediators Targeting Inflammation

**DOI:** 10.3389/fphys.2021.640374

**Published:** 2021-07-15

**Authors:** Bohae Rachel Lee, May Hnin Paing, Neelam Sharma-Walia

**Affiliations:** H. M. Bligh Cancer Research Laboratories, Department of Microbiology and Immunology, Chicago Medical School, Rosalind Franklin University of Medicine and Science, North Chicago, IL, United States

**Keywords:** prostaglandins, PPAR-γ, viral (or virus), inflammation, antiviral

## Abstract

Cyclopentenone prostaglandins (cyPGs) are biologically active lipid mediators, including PGA_2_, PGA_1_, PGJ_2_, and its metabolites. cyPGs are essential regulators of inflammation, cell proliferation, apoptosis, angiogenesis, cell migration, and stem cell activity. cyPGs biologically act on multiple cellular targets, including transcription factors and signal transduction pathways. cyPGs regulate the inflammatory response by interfering with NF-κB, AP-1, MAPK, and JAK/STAT signaling pathways via both a group of nuclear receptor peroxisome proliferator-activated receptor-gamma (PPAR-γ) dependent and PPAR-γ independent mechanisms. cyPGs promote the resolution of chronic inflammation associated with cancers and pathogen (bacterial, viral, and parasitic) infection. cyPGs exhibit potent effects on viral infections by repressing viral protein synthesis, altering viral protein glycosylation, inhibiting virus transmission, and reducing virus-induced inflammation. We summarize their anti-proliferative, pro-apoptotic, cytoprotective, antioxidant, anti-angiogenic, anti-inflammatory, pro-resolution, and anti-metastatic potential. These properties render them unique therapeutic value, especially in resolving inflammation and could be used in adjunct with other existing therapies. We also discuss other α, β -unsaturated carbonyl lipids and cyPGs like isoprostanes (IsoPs) compounds.

## Introduction

Prostaglandins (PGs) are a group of lipids or oxygenated derivatives of arachidonic acid (AA) that sustain homeostatic functions and mediate the inflammatory response (Aoki and Narumiya, 2012). There are two types of PGs: conventional or classic PGs and cyclopentenone PGs (cyPGs). Examples of traditional PGs are PGD_2_, PGE_2_, prostacyclin (PGI_2_), PGF_2__α_, and thromboxane A_2_ (TXA_2_), while the members of cyPGs include PGA_1_, PGA_2_, PGJ_2_, and metabolites of PGJ_2_, such as 15-Deoxy-Δ-^12,14^-Prostaglandin J_2_ (15d-PGJ_2_) and Δ^12^-PGJ_2_. As the name implies, cyPGs contain a cyclopentenone ring structure with a highly reactive α, β-unsaturated carbonyl group, which can alter many proteins and their functional properties covalent attachments with thiol groups of the proteins (Straus and Glass, 2001). cyPGs are potent bioactive molecules and have a wide range of functions (Burstein, 2020). cyPGs can repress inflammatory responses, inhibit cell growth, angiogenesis, and increase apoptosis. cyPGs can interfere with virus infections and cancer development, indicating their potential to serve as therapeutic agents. This review discusses cyPGs biosynthesis, mechanism of action, functions, and their effects on virus infection and cancer development. Despite the existing knowledge, the resolving, antiviral, anti-inflammatory, and anticancer potential of cyPGs have been minimally explored and warrant further attention.

## Biosynthesis of Cyclopentenone Prostaglandins (PGA_1_, PGA_2_, and PGJ_2_ and Its Metabolites)

**FIGURE 1:**
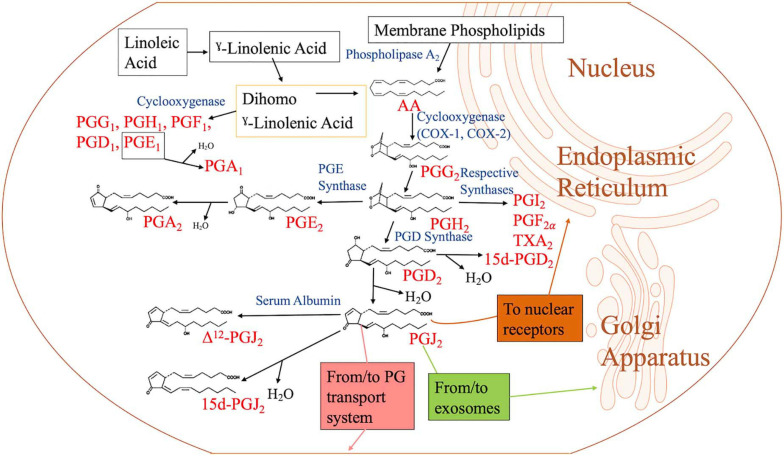
Biosynthesis of cyclopentenone prostaglandins. When the cell is activated by stressful stimuli, such as mechanical trauma, interferon, interleukin, or growth factors, the enzyme phospholipase A_2_ moves from the cytoplasm to intracellular membranes to liberate arachidonic acid (AA) from the nuclear envelope or endoplasmic reticulum. AA is converted by cyclooxygenase-1 (COX-1) or cyclooxygenase-2 (COX-2) to prostaglandin G_2_ (PGG_2_), followed by hydroperoxidation of PGG_2_ to PGH_2_. PGH_2_ is converted to other PGH_2_ metabolites such as PGD_2_, PGE_2_, PGF_2_, PGI_2_, and thromboxane A_2_ (TXA_2_) by their respective synthases. Of the metabolites, PGD_2_ is dehydrated to form J_2_ PGs. PGJ_2_ may be located in exosomes, transport systems, or nuclear receptors to execute its function. PGE_2_ is dehydrated to form PGA_2_. PGA_1_ is a metabolite of linoleic acid, which is obtained through diet.

AA is liberated from membrane phospholipids by the enzyme phospholipase A_2_ (PLA_2_) (Vane and Botting, 1990). Myosin, an actin-binding protein, is phosphorylated when there is an increase in intracellular calcium levels, causing PLA_2_ to translocate from the cytoplasm to the intracellular membrane to access the phospholipids. Arachidonate is metabolized to PGG_2_ by cyclooxygenase (COX) 1 and 2 (COX-1 and COX-2), which are contained in the endoplasmic reticulum (ER) and nuclear membranes (Vane and Botting, 1990; Hanna and Hafez, 2018) (Figure 1). PGG_2_ is converted into PGH_2_ by hydroxyperoxidase. Unstable PGH_2_ diffuses from the ER lumen to the cytoplasm through the ER membrane. Due to its unstable nature, PGH_2_ is enzymatically converted into different PGs, including PGI_2_, PGF_2_, and TXA_2_, through the action of specific PG synthases (Figure 1). When PGH_2_ is acted upon by PGD_2_ synthase, PGD_2_ is created. PGD_2_ is unstable and spontaneously undergoes non-enzymatic dehydration to yield either 15d-PGD_2_ or PGJ_2_ (Figure 1). Further dehydration and a 13, 14 double bond rearrangement of PGJ_2_ yield 15-Deoxy-Δ-^12,14^-prostaglandin J_2_ (15d-PGJ_2_) in an albumin-independent manner, while PGJ_2_ dependent on serum albumin results in Δ12-PGJ_2_ (Figueiredo-Pereira et al., 2014). PGs of the J series are synthesized *in vivo* as Δ12-PGJ_2_ is a natural component of human body fluids. Its synthesis is inhibited by treatment with COX inhibitors (Hirata et al., 1988). When PGH_2_ is acted upon by PGE_2_ synthase, PGE_2_ is formed. Dehydration of PGE_2_ leads to PGA_2_ (Hamberg and Samuelsson, 1966; Nugteren et al., 1966) (Figure 1). 15d-PGJ_2_ could function in both an autocrine and paracrine manner and can be produced intracellularly and extracellularly via non-enzymatic conversion of PGD_2_ (Shibata et al., 2002).

The formation of the cyclopentenone PGA_1_ has a different genesis pathway compared to the other members of its family (PGA_2_ and PGJ_2_). The formation of PGA_1_ begins with linoleic acid (LA). In the human diet, linoleic acid is the most consumed polyunsaturated fatty acid (PUFA) (Whelan and Fritsche, 2013). Linoleic acid, an essential omega 6 (*n* = 6) fatty acid, is converted to γ-linoleic acid (GLA; GLA, 18:3-6) through the membrane-bound enzyme 6-desaturase (Δ-6-desaturase). GLA is then metabolized to dihomo γ-linolenic acid (DGLA, 20:3-6) by a Δ6 elongase. From this point, DGLA can be converted into AA by the enzyme 5-Desaturase, or PGE_1_ by the enzyme COX. PGE_1_ undergoes dehydration to become PGA_1_ (Kapoor and Huang, 2006; Kapoor et al., 2007).

15d-PGJ_2_ acts via G-protein-coupled seven-transmembrane PGD_2_ receptors (D prostanoid; DP_1_ and DP_2_) and through interaction with intracellular targets (Kato et al., 1986; Kim et al., 1993; Negishi and Katoh, 2002). DP_2_ (chemoattractant receptor-homologous molecule or GPR44 or CD294) is expressed on Th2 cells, eosinophils, activated mast cells, and basophils (Negishi and Katoh, 2002; Nagata et al., 2017). PGE_1_/PGA_1_ is native/endogenous ligands of orphan nuclear receptor-related 1 protein (Nurr1; NR4A2) and activates its transcriptional function (Negishi and Katoh, 2002; Pearen and Muscat, 2010; Kurakula et al., 2014).

## Cyclopentenone Prostaglandins and Inflammation

### Cyclopentenone Prostaglandins in Various Diseases

15d-PGJ_2_ is an immune regulator to modulate human autoimmune diseases as multiple sclerosis (MS), experimental allergic encephalomyelitis (EAE), polymyositis, Bechet’s diseases, rheumatoid arthritis (RA), atopic dermatitis, systemic lupus erythematosus (SLE) (Li and Pauza, 2009), and age-related neurodegenerative diseases, including Alzheimer’s (AD) and Parkinson’s disease (PD) (Koharudin et al., 2011). γΔT cells have been studied in context with autoimmune diseases in humans. γΔT cells possess the cytotoxic activity and produce IFN-γ, tumor necrosis factor-alpha; TNF-α, and chemokines involved in recruiting monocytes and macrophages. The induction of cytokines and secretion of interleukin-17 (IL-17) contributes to inflammatory processes and promotes autoimmunity. 15d-PGJ_2_, along with rosiglitazone (Avandia), suppressed γΔT cell proliferation in response and downregulated cytokine production (Li and Pauza, 2009). 15d-PGJ_2_ also plays an essential regulatory role in osteosarcoma, bone metastases, and bone metabolism (Kitz et al., 2011; Kim et al., 2015).

### Cyclopentenone Prostaglandins Elicit Anti-inflammatory Responses via Regulating Transcription Factors Crucial for Inflammatory Response

15d-PGJ_2_ directly inhibits multiple steps in the NF-κB signaling pathway and NF-κB-dependent gene expression (Straus et al., 2000). NF-κB represents a family of structurally related inducible transcription factors (NF-κB1; p50, NF-κB2; p52, RelA; p65, RelB, and c-Rel) located in the cytoplasm, which activates genes responsible for inflammation and innate and adaptive immunity (Senftleben et al., 2001). The NF-κB proteins are typically sequestered in the cytoplasm by a family of inhibitory proteins, including IκB family members, which sterically block the nuclear localization sequence (NLS) of NF-κB (Senftleben et al., 2001; Sun, 2017). The IκB kinase (IKK) complex is crucial for the activation of NF-κB, as it can degrade the NF-κB inhibitor IκB through phosphorylation, subsequently freeing NF-κB (Senftleben et al., 2001). NF-κB is involved in the pathogenesis of inflammatory diseases, including RA, inflammatory bowel disease (IBD), MS, atherosclerosis, SLE, type 1 diabetes, chronic obstructive pulmonary disease (COPD), and asthma (Pai and Thomas, 2008). NF-κB activation induces proinflammatory cytokines (IL-1β, IL-1, IL-2, IL-6, IL-8, and TNF-α) (Lawrence, 2009; Wang et al., 2014) and regulates inflammasome function (Guo et al., 2015) in both innate and adaptive immune cells. PGA_1_, another cyPG, is also a potent inhibitor of NF-κB activation in human cells by inhibiting phosphorylation and preventing degradation of the NF-κB inhibitor IκB-α (Rossi et al., 1997). The α, β-unsaturated carbonyl group in the cyPGs, when reactive, can undergo a Michael reaction with the cysteine nucleophile at position 179 on the IKKβ subunit of the IKK complex. This cysteine is located in the activation loop of the enzyme, and the alkylation of the cysteine inhibits the phosphorylation of the activation loop. Therefore, cyPGs inhibit IKK complex activity by directly modifying the IKKβ subunit (Rossi et al., 2000). By doing so, the degradation IκB is inhibited, and NF-κB is unable to enter the nucleus.

15d-PGJ_2_ inhibits transcription factor activity of activating protein-1 (AP-1) (Perez-Sala et al., 2003). AP-1 is composed of dimeric complexes, which included members of four families of DNA-binding proteins such as Jun, Fos, ATF/cyclic AMP-responsive element-binding (CREB), and musculoaponeurotic fibrosarcoma (Maf) (Milde-Langosch, 2005; Hernandez et al., 2008). 15d-PGJ_2_ covalently modifies c-Jun and directly inhibits the DNA binding activity of AP-1 (Perez-Sala et al., 2003). AP-1 plays critical roles in inflammation, proliferation, innate immune response and stimulates growth factors and proinflammatory cytokines mediated by serine/threonine kinases as c-Jun NH2-terminal kinases (JNK), p38, extracellular signal-regulated kinases (ERK), and c-Fos-regulating kinases (FRK) MAP kinase pathways (Lin et al., 1995; Minden et al., 1995).

15d-PGJ_2_ non-specifically inhibits Signal transducer and activator of transcription (STAT) (Ji et al., 2005) and Janus kinase (JAK)-STAT signaling pathway in lymphocytes (Kim et al., 2005). STAT1 can be activated upon tyrosine phosphorylation by JAK1 tyrosine kinase (Mowen and David, 2000). Upon activation, STAT/STAT interactions occur immediately, and dimerized STATs can then enter the nucleus and regulate the transcription of inflammatory genes of cytokine and interferon signaling (Seif et al., 2017).

### Anti-inflammatory, Anti-tumorigenic, Anti-angiogenic, Anti-metastatic, Anti-fibrotic, Resolving, and Antioxidant Modes of Action of Cyclopentenone Prostaglandins

cyPGs, such as 15d-PGJ_2_, PGJ_2_, PGA_1_, and PGA_2_, can activate peroxisome proliferator-activated receptor-gamma (PPAR-γ), and many of their biological functions are either PPAR-γ dependent or independent (Mukherjee et al., 1994; Ricote et al., 1998b; Yagami et al., 2018). PPAR-γ is one of the members (PPAR-α, PPAR-δ, and PPAR-γ) of the nuclear receptor superfamily and is a ligand-dependent transcription factor. The ligand 15d-PGJ_2_ activates PPAR-γ, and PPAR-γ then forms a heterodimer with retinoid X receptor (RXR) in the cytoplasm. Complex enters the nucleus (Scher and Pillinger, 2005; Li et al., 2019). This complex binds to specific PPAR response element (PPRE) regions in the DNA to activate different target genes (Forman et al., 1996).

#### Anti-inflammatory Actions

Peroxisome proliferator-activated receptor-gamma inhibits TNF-α, IL-6, inducible NO synthase (iNOS), gelatinase B, and COX-2 by acting as an antagonist to AP-1 and NF-κB (Welch et al., 2003). This inhibition mode was observed in activated macrophages expressing high levels of PPAR-γ (Ricote et al., 1998a,b; Straus et al., 2000). In general, when IFN-γ stimulated peritoneal macrophages were treated with 15d-PGJ_2_, instead of observing activated macrophages, morphological features classic of resting cells were seen (Ricote et al., 1998a,b). 15d-PGJ_2_ treatment inhibited the induction of iNOS transcription by inhibiting the binding of AP-1 and NF-κB on iNOS promoter (Ricote et al., 1998a,b). Usually, iNOS is upregulated in activated macrophages accompanied by the overproduction of nitric oxide (NO), which causes inflammation (Sharma and Staels, 2007). Excess NO also induces *s*-nitrosylation of Sirt1, an inhibitor of p65 NF-κB, which inactivates Sirt1 and enhances pro-inflammatory response (Nakazawa et al., 2017). 15d-PGJ_2_ treatment inhibits matrix metalloproteinase (MMP-9) or also called Gelatinase B in activated macrophages (Ricote et al., 1998a,b) at the transcription level. Inhibition by 15d-PGJ_2_ is mediated at the level of AP-1 binding as MMP-9 transcriptional activation is dependent on AP-1 (Saarialho-Kere et al., 1993). 15d-PGJ_2_ and TZDs reduced dendritic cells (DCs) stimulation with toll-like receptor (TLR) ligands via the MAP kinase and NF-κB pathways (Appel et al., 2005). In RAW264.7 cells, monocyte/macrophage-like cell lineage stimulated with LPS, a similar outcome to that of Jurkat cells was observed when treated with cyPGs (Straus et al., 2000). A different result was observed in HeLa cells, strengthening the fact that cyPGs’ effect is cell type specific. Instead of inhibiting IKK complex activity, cyPGs impede the binding of NF-κB to DNA since p50 and p65 have cysteine residues at C62 and C38, respectively. Alkylation of these cysteines via the Michael reaction results in the inhibition of the binding of NF-κB to DNA (Straus et al., 2000).

In human astrocytes treated with 15d-PGJ_2_, NF-κB was inhibited from binding to the COX-2 promoter on DNA (Janabi, 2002). In glial cells, 15d-PGJ_2_ induces the transcription of suppressor of cytokine signaling 1 and 3 (SOCS1 and SOCS3) can inhibit JAK, eventually inhibiting the transcription of inflammatory genes (Park E. J. et al., 2003; Park S. H. et al., 2003). 15d-PGJ_2_ inhibited the JAK/STAT1 mediated interferon regulatory factor-1 (IRF-1) expression, thereby decreasing the IFN-γ-induced costimulatory molecule B7-H1 expression needed by tumors to evade the host immune response (Seo et al., 2014). 15d-PGJ_2_ inhibits lethal anthrax toxin (LT) activation of the NLRP1 and nigericin-mediated activation of the NLRP3 inflammasome and associated IL-1β release (Maier et al., 2015). 15d-PGJ_2_ mitigates the macrophage hyperinflammatory response (Monroy et al., 2007).

PGD_2_ and the J_2_-series PGJ_2_ and Δ12-PGJ_2_ are critical components of the inflammatory response within adipose tissue during obesity thus producing inflammation-related adipokines implicated in insulin sensitivity (Peeraully et al., 2006). 15d-PGJ_2_ is the most potent inducer of fat cell (adipocyte) differentiation *in vitro* (Forman et al., 1995; Bell-Parikh et al., 2003). PGD_2_, PGJ_2_, and Δ12-PGJ_2_ treatment strongly down-regulates the production of leptin, a hormone secreted by adipocytes (Peeraully et al., 2006).

#### Anti-tumorigenic Actions

15d-PGJ_2_ exerts antitumor activity by regulating the Myc/Mad/max transcription factors to promote cell apoptosis, tubulin binding activity, inhibiting the expression of human telomerase reverse transcriptase (hTERT), enhancing TRAIL-induced apoptosis by downregulating AKT phosphorylation, reactive oxygen species (ROS)-dependent cell death pathway, ROS-dependent AKT activation, inhibition of COX-2, STAT-3, cell cycle (G2/M or G1) blockade, inhibition of vascular endothelial factor (VEGF), growth and expansion of tumor stem cells in gastric cancer (Inoue et al., 2000; Sato et al., 2000; Takashima et al., 2001; Yuan et al., 2005; Chearwae and Bright, 2008; Dionne et al., 2010; Li et al., 2017), oral squamous cell carcinoma (Nikitakis et al., 2002), leukemia (Han et al., 2007), lymphoma (Inoue et al., 2000; Sato et al., 2000; Takashima et al., 2001; Yuan et al., 2005; Chearwae and Bright, 2008; Dionne et al., 2010; Li et al., 2017), oesophageal cancer (Takashima et al., 2001), endometrial cancer (Li and Narahara, 2013), breast cancer (Cocca et al., 2009), osteosarcoma (Yen et al., 2014), and brain tumors (Inoue et al., 2000; Sato et al., 2000; Takashima et al., 2001; Yuan et al., 2005; Chearwae and Bright, 2008; Dionne et al., 2010; Li et al., 2017) (Table 1). Transforming growth factor-β (TGF-β) induces cell growth, cell migration, and epithelial to mesenchymal transition (EMT) and promotes HCC progression (Giannelli et al., 2014). Interestingly, TZDs and 15d-PGJ_2_ display antitumor effects on HCC (Hsu and Chi, 2014). PPAR-γ activation inhibits TGF-β expression via dephosphorylation of zinc finger transcription factor-9 (Zf9) (Lee et al., 2006). Zf9 is crucial for TGFβ1 gene regulation, and a phosphorylated form of Zf9 transactivates the TGFβ1 promoter (Kim et al., 1998).

**TABLE 1:** Biological effects of cyclopentenone prostaglandins.

Anti-inflammatory	Specific function	Site of action	References
15d-PGJ_2_	Inhibition of iNOS promoter containing binding sites for AP-1 and NF-κB	Macrophages	Ricote et al., 1998a,b
15d-PGJ_2_	Gelatinase B or MMP-9	Macrophages	Ricote et al., 1998a,b
15d-PGJ_2_ and TZDs	MAPK and NF-κB signaling	Dendritic cells (DCs)	Appel et al., 2005
15d-PGJ_2_, other cyPGs	Inhibition of NF-κB binding to DNA	RAW264.7 cells, monocyte/macrophage-like cell lineage	Straus et al., 2000
15d-PGJ_2_	Inhibition of NF-κB binding to the COX-2 promoter STAT-1 and c-Jun expression	Human astrocytes, microglia	Janabi, 2002
15d-PGJ_2_	Transcription of SOCS1 and SOCS3	Brain inflammation	Park E. J. et al., 2003; Park S. H. et al., 2003
15d-PGJ_2_	Inhibition of the JAK/STAT1 mediated IRF-1 expression decreasing cytokine production	B16F10 melanoma cells	Seo et al., 2014
15d-PGJ_2_	Inhibition of caspase-1 activation by NLRP1 and NLRP3 inflammasomes prevents the autoproteolytic activation of caspase-1 and the maturation of IL-1β	NLRP3-dependent peritonitis model	Maier et al., 2015
15d-PGJ_2_	Mitigates the macrophage hyperinflammatory response and inflammatory cytokines	Macrophages	Monroy et al., 2007
PGD_2_, PGJ_2_, and Δ12-PGJ_2_	Down-regulate the production of leptin	3T3-L1 adipocytes	Peeraully et al., 2006
15d-PGJ_2_	Inhibition of NF-κB signaling and at PI3K/Akt pathway	Primary astrocytes	Giri et al., 2004
PGA_1_, PGJ_2_, PGD and 15d-PGJ_2_	Direct inhibition, and modification of the IKKβ subunit, improve the utility of COX2 inhibitors.	Jurkat cells (immortalized line of human T lymphocyte cells)	Rossi et al., 2000
**Anti-tumorigenic**			
15d-PGJ_2_	Myc/Mad/max transcription factors	Gastric cancer, Oral Squamous cell carcinoma, Leukemia, Lymphoma, Oesophageal cancer, Endometrial cancer, Breast cancer, and Brain tumors	Inoue et al., 2000; Sato et al., 2000; Takashima et al., 2001; Nikitakis et al., 2002; Yuan et al., 2005; Han et al., 2007; Chearwae and Bright, 2008; Cocca et al., 2009; Dionne et al., 2010; Li and Narahara, 2013; Li et al., 2017
15d-PGJ_2_	Enhancing TRAIL-induced apoptosis by downregulating AKT expression and phosphorylation	Leukemia	Han et al., 2007
15d-PGJ_2_	ROS-dependent AKT activation, cell cycle inhibition	Osteosarcoma	Yen et al., 2014
15d-PGJ_2_	A tubulin-binding agent that destabilizes microtubules and induces mitotic arrest	Breast cancer	Cocca et al., 2009
15d-PGJ_2_	Cell cycle blockade	Oesophageal cancer	Takashima et al., 2001
15d-PGJ_2_ and TZDs	Tumor cell growth, migration, and invasion	Hepatocellular carcinoma (HCC)	Hsu and Chi, 2014
15d-PGJ_2_ and its derivatives (J11-C1)	Expression of genes associated with cell cycle arrest, apoptosis, and autophagy, decreased expression of the anti-apoptotic Bcl-2	Ovarian cancer SKOV3 cells	Tae et al., 2018
15d-PGJ_2_	Inhibition of STAT-3	Oral Squamous cell carcinoma	Nikitakis et al., 2002
15d-PGJ_2_	Apoptosis rate, Apoptosis-promoting protein, and reduced apoptosis-inhibiting protein expression	Hepatitis B virus (HBV) × protein (HBx)-positive HL7702-HBx and HL7702 liver cells	Chen et al., 2014
**Anti-angiogenic Anti-metastatic**			
15d-PGJ_2_ Pioglitazone	Inhibiting VEGF	Renal cell carcinoma (RCC)	Yuan et al., 2005
15d-PGJ_2_	Inhibiting angiopoietin-1 (Ang-1)	Gastric cancer	Fu et al., 2006
15d-PGJ_2_	Reduced VEGF receptor 1 (Flt-1) and 2 (Flk/KDR), urokinase plasminogen activator (uPA), and increased plasminogen activator inhibitor-1 (PAI-1) mRNA	Human umbilical vein endothelial cells (HUVEC)	Xin et al., 1999; Funovics et al., 2006
15d-PGJ_2_ (PPAR-γ dependent), BRL49653, Ciglitizone	Block angiogenesis	Rat cornea	Xin et al., 1999
15d-PGJ_2_ HO-1-dependent mechanism	NF-κB and AP-1 mediated MMP-9 expression and invasion	MCF-7 breast cancer cells	Jang et al., 2020
15d-PGJ_2_	Disassembled focal adhesions, downregulation of FAK signaling	Renal cell carcinoma (RCC) metastasis	Yamamoto et al., 2020
**Antioxidant**			
15d-PGJ_2_	Nrf2-Keap1 signaling pathway	Atherosclerosis	Itoh et al., 2004; Levonen et al., 2004; Mochizuki et al., 2005
15d-PGJ_2_	HO-1, SOD, catalase, NAD(P)H dehydrogenase quinone 1 (NQO1), c-glutamylcysteine synthetase (GCS), glutathione reductase (GR), glutathione peroxidase 1 (GPx)	Pleurisy, atherosclerosis	Diers et al., 2010; Itoh et al., 2004; Kansanen et al., 2009; Magesh et al., 2012
15d-PGJ_2_	15-PGDH gene expression, protein level, and its activity, AP-1 and HO-1	Human colon cancer cell line HCT-116	Park and Na, 2019a,b; Tauber and Parker, 2019
15d-PGJ_2_	eIF2α phosphorylation, Activation of Integrated stress response (ISR)	Neurodegenerative diseases	Park and Na, 2019a,b; Tauber and Parker, 2019
**Resolving inflammation**			
15d-PGJ_2_	Cytoprotective, Shifting PG production from PGE_2_ to PGD_2_ and 15d-PGJ_2_	Dextran sodium sulfate-induced colitis in the rat and TNF-α-induced activation of PG production and PG synthase expression in cultured human peripheral blood monocytes (hPBMC)	Niro et al., 2010
15d-PGJ_2_	DP1 receptor activation checkpoint controller of cytokine/chemokine synthesis as well as leukocyte influx and efflux	Self-resolving peritonitis	Rajakariar et al., 2007
15d-PGJ_2_	PPAR-γ and CD36 expression	Enhance hematoma resolution	Flores et al., 2016
15d-PGJ_2_	Inhibition of pro-inflammatory cytokines, such as IL-5, IL-13, IL-17, TNF-α Inhibition of NF-κB phosphorylation	Peribronchial accumulation of eosinophils and neutrophils, subepithelial fibrosis, and also mucus exacerbation	Coutinho et al., 2017
**Prostanylation and protein modification**			
PGE_1_ and PGA_1_	Interact with the ligand-binding domain (LBD) of orphan nuclear receptor Nurr1, neuroprotective, enhanced expression of Nurr1 target genes in midbrain dopaminergic (mDA) neurons and improved motor deficits	Mouse models of Parkinson’s disease	Rajan et al., 2020
15d-PGJ_2_ and PGA_1_	IKKα and β, NF-κB P65 and P50 subunits cysteine modification at various positions	Inhibition of NF-κB pathway	Castrillo et al., 2000; Rossi et al., 2000; Cernuda-Morollon et al., 2001
15d-PGJ_2_ and PGA_1_	H-Ras modification at various cysteines	Activation of H-Ras	Oliva et al., 2003
15d-PGJ_2_	c-Jun and c-Fos modification at various cysteines	Inhibition	Perez-Sala et al., 2003
PGA_1_	Thioredoxin, thioredoxin reductase, and Keap1	Inhibition	Levonen et al., 2001, 2004; Shibata et al., 2003a; Itoh et al., 2004
15d-PGJ_2_	Proteasome	Inhibition	Shibata et al., 2003b

15d-PGJ_2_ and its derivatives exert antitumor activity by selectively modulating the expression of genes associated with cell cycle arrest, apoptosis, and autophagy (Inoue et al., 2000; Sato et al., 2000; Takashima et al., 2001; Yuan et al., 2005; Chearwae and Bright, 2008; Dionne et al., 2010; Li et al., 2017). Notably, J11-C1 is a novel candidate of class III histone deacetylases (HDACs) called Sirtuin SIRT1 inhibitor with anticancer activity. SIRTs are involved in biological functions, including aging, energy mobilization, and stress responses. SIRTs regulate cancer cell apoptosis and are potential targets for novel anticancer drugs that regulate the levels of deacetylated histone proteins, p53, and several transcriptional factors (Table 1) (Tae et al., 2018). 15d-PGJ_2_ treatment significantly induced apoptosis rate, apoptosis-promoting protein expression, and reduced apoptosis-inhibiting protein expression in the hepatitis B virus (HBV) × protein (HBx)-positive HL7702-HBx and HL7702 liver cells (Chen et al., 2014).

#### Anti-angiogenic/Anti-metastatic Actions

15d-PGJ_2_ exerts anti-angiogenic activity by inhibiting VEGF and angiopoietin-1 (Ang-1) in renal cancer (Yuan et al., 2005) and gastric cancer (Fu et al., 2006), respectively. Treatment of human umbilical vein endothelial cells (HUVEC) with 15d-PGJ_2_ reduced mRNA levels of VEGF receptors 1 (Flt-1) and 2 (Flk/KDR) and urokinase plasminogen activator (uPA) and increased plasminogen activator inhibitor-1 (PAI-1) mRNA (Funovics et al., 2006). Administration of 15d-PGJ_2_ could inhibit VEGF-induced angiogenesis in the rat cornea in a PPAR-γ dependent manner (Xin et al., 1999) (Table 1). Rosiglitazone (Avandia) and troglitazone (TGZ) inhibit cell migration via the upregulation of E-cadherin expression in HepG2 cells (Lee et al., 2009). 15d-PGJ_2_ inhibits NF-κB and AP-1-mediated MMP-9 expression and invasion of MCF-7 breast cancer cells employing a heme oxygenase-1 (HO-1)-dependent mechanism (Jang et al., 2020). Treatment with a low concentration of 15d-PGJ_2_ disassembled focal adhesions, reduced focal adhesion kinase (FAK) phosphorylation, and caused extensive filamentous actin reorganization (Yamamoto et al., 2020). PPAR-γ did not mediate the inhibitory effect of 15d-PGJ_2_ on the migration of Caki-2 cells and did not affect RCC metastasis (Yamamoto et al., 2020).

#### Antioxidant and Resolving Actions

Inflammation is accompanied by the production of ROS, and 15d-PGJ_2_ has antioxidant properties (Itoh et al., 2004; Levonen et al., 2004; Mochizuki et al., 2005) (Table 1). 15d-PGJ_2_ and structurally related isoprostanoids alkylate Kelch-like ECH-associated protein 1 (Keap1) to induce the NF-E2-related nuclear factor erythroid-2 (Nrf2-) dependent antioxidant bioactivity (Levonen et al., 2004; Kansanen et al., 2009; Diers et al., 2010; Mills et al., 2018). 15d-PGJ_2_ activates Nrf2-Keap1 signaling and induces gene transcription of antioxidant enzymes including HO-1, superoxide dismutase (SOD), catalase, NAD(P)H dehydrogenase quinone 1 (NQO1), c-glutamylcysteine synthetase (GCS), glutathione reductase (GR), and glutathione peroxidase 1 (GPx) (Itoh et al., 2004; Kansanen et al., 2009; Diers et al., 2010; Magesh et al., 2012). 15d-PGJ_2_ upregulates 15−hydroxyprostaglandin dehydrogenase (15-PGDH) gene expression, protein level, and its activity in human colon cancer cell line HCT-116 through AP-1 activation (Park and Na, 2019a,b). 15d-PGJ_2_ treatment-induces eIF2α phosphorylation and activation of the integrated stress response (ISR), also leading to bulk translation repression and preferential translation of stress response mRNAs (Tauber and Parker, 2019). 15d-PGJ_2_ is pro-resolving signaling and a neuroprotective (Rajan et al., 2020) molecule (Table 1) (Rajakariar et al., 2007; Niro et al., 2010; Flores et al., 2016; Coutinho et al., 2017).

### Pro-metastatic Properties of the Cyclopenenone Prostaglandins

cyPGs also exhibit pro-metastatic properties such as 15d-PGJ_2_ significantly enhanced the rate of formation, the size, and the vascularization of papillomas in a murine carcinogenesis model (Millan et al., 2006). 15d-PGJ_2_ and PGJ_2_ induced the proliferation of COX-2 depleted colorectal cancer (HCA-7) cells at a nanomolar concentration (Chinery et al., 1999). However, the precise mechanisms responsible for tumor proliferative effects of 15d-PGJ_2_ remain incompletely clarified. VEGF is well known as a master regulator of angiogenic switch (Bussolati and Mason, 2006). Interestingly, VEGF upregulates HO-1 in vascular endothelial cells, while HO-1 may also regulate the synthesis and activity of VEGF, thus constituting a positive feedback loop (Bussolati and Mason, 2006). 15d-PGJ_2_ could stimulate VEGF expression in endothelial cells, human androgen-independent PC3 prostate cancer cells, and the 5,637 urinary bladder carcinoma cell line (Yamakawa et al., 2000; Haslmayer et al., 2002). The upregulation of VEGF by 15d-PGJ_2_ was accompanied by activation of PPAR-γ (Jozkowicz et al., 2002). However, the VEGF promoter does not harbor PPRE (Inoue et al., 2001; Jozkowicz et al., 2004). Interestingly, VEGF upregulation by 15d-PGJ_2_ could be mimicked by the induction of HO-1 expression (Jozkowicz et al., 2004). 15d-PGJ_2_ induced HO-1 expression in MCF-7 human breast cancer cells (Kim et al., 2004).

Nrf2, a transcription factor is responsible for maintenance of cellular redox balance (Loboda et al., 2016). HO-1 is a prototypic Nrf2 target gene, and the aberrant hyperactivation of Nrf2/HO-1 axis contributes to tumor progression, aggressiveness, chemoresistance, and poor prognosis (Zimta et al., 2019). 15d-PGJ_2_ induces VEGF expression and angiogenesis in human breast cancer cells through upregulation of HO-1 (Kim et al., 2006; Kweider et al., 2011).

## Role of Cyclopentenone Prostaglandins During Viral Infections

### Cyclopentenone Prostaglandins as Inhibitor of Viral Replication

cyPGs are potent inhibitors of viral replication (Table 2) and are effective against a wide range of viruses. These include negative-strand RNA viruses such as influenza A (Pica et al., 1993, 2000; Conti et al., 2001), Sendai virus (Amici and Santoro, 1991; Amici et al., 2001), and vesicular stomatitis virus (VSV) (Santoro et al., 1987; Pica et al., 1993); positive-strand RNA viruses such as Sindbis virus (Mastromarino et al., 1993), Poliovirus (Conti et al., 1996), and Human immunodeficiency virus-1 (Rozera et al., 1996) and DNA viruses such as herpes simplex virus (HSV) type 1 and 2 (Yamamoto et al., 1987; Amici et al., 2001). The ability of cyPGs to suppress virus production is very dramatic. In the African green monkey kidney (AGMK) cell line, replication of the Sendai virus is almost completely inhibited by 4 mg/ml of PGA_1_ (Santoro et al., 1987) and by 4 mg/ml of PGJ_2_ (Santoro et al., 1987) without being toxic to uninfected AGMK cells. Treatment of 6 mg/ml of Δ^12^-PGJ_2_ in Madin–Darby canine kidney cells (MDCK) infected with influenza A H1N1 (PR8) virus drastically suppressed the viral production by 95%. Simultaneously, a higher dose of Δ^12^-PGJ_2_ produced an undetectable virus yield (Pica et al., 1993). PGA_1_ treatment also strongly inhibits the viral production of Ulster 73 (H7N1 influenza A) in LLC-monkey kidney epithelial cells (LLC-MK2), African green monkey kidney-37RC cells (AGMK-37RC), and MDCK cells (Conti et al., 2001), suggesting that cyPGs are effective against various subtypes of influenza A virus in multiple host cells. Similarly, *in vivo* studies have shown that PGA_1_ and 16, 16-dimethyl-PGA_2_ (dmPGA_2_), a long-acting synthetic analog of PGA, in mice infected with a lethal dose of PR8 virus significantly decreases the virus titers in the lung and increases the survival rate (Santoro et al., 1987; Pica et al., 1993). In another study, the antiviral activity of the synthetic dmPGA_1_ in HSV-1 and human immunodeficiency virus (HIV)- infected cells was investigated (Hughes-Fulford et al., 1992). dmPGA_1_ affected HIV-1 replication in acutely infected T cells and chronically infected macrophages as assessed by a quantitative decrease in HIV-1 antigen p24 concentration (Hughes-Fulford et al., 1992). This study highlighted the unusual broad-spectrum antiviral activity of dmPGA_1_ against HSV and HIV-1 and its therapeutic potential for *in vivo* use (Hughes-Fulford et al., 1992).

**TABLE 2:** Effects of cyclopentenone prostaglandins in viral infections.

Anti-viral Activity	Virus	CyPGs	Mechanism	References
**Inhibition of virus replication by altering viral gene/protein expression (transcription/translation level alteration)**	Influenza A	Δ^12^-PGJ_2_	Decrease synthesis of hemagglutinin (HA), nucleoprotein (NP), and membrane protein M1; induction of 70 kDa host HSP70	Pica et al., 1993
		PGA_1_	Delayed synthesis of HA, membrane protein M1, structural protein M2, and non-structural protein NS2; induction of 70 kDa host HSP70	Conti et al., 2001
	Vesicular Stomatitis Virus (VSV)	Δ^12^-PGJ_2_, PGA_1_	Inhibit VSV RNA polymerase	Bader and Ankel, 1990; Pica et al., 1993; Parker, 1995
	Herpes Simplex Virus Type 1 (HSV-1)	PGA_1_	Suppress NF-κB activation by inhibiting IKK complex (independent of the PPAR-γ pathway)	Amici et al., 2001
	Herpes Simplex Virus Type 2 (HSV-2)	Δ7-PGA_1_, Δ^12^-PGJ_2_	Inhibited the primary transcription of HSV-2	Yamamoto et al., 1987
	Human Immunodeficiency Virus-1 (HIV-1)	PGJ_2_	Suppress NF-κB activation by inhibiting IKK complex (independent of the PPAR-γ pathway)	Rozera et al., 1996; Boisvert et al., 2008
		15d-PGJ_2_	Covalently modify HIV-1 transactivating protein, Tat to inhibit virus transcriptional elongation	Kalantari et al., 2009
**Inhibition of virus replication by altering viral glycoprotein glycosylation (post-translational level alteration)**	Vesicular Stomatitis Virus (VSV)	Δ^12^-PGJ_2_	Inhibit glycosylation of virus glycoprotein G	Pica et al., 1993
	Sendai Virus	PGA_1_, Δ^12^-PGJ_2_	Inhibit glycosylation of virus glycoproteins hemagglutinin-neuraminidase (HN) and fusion protein (F)	Santoro et al., 1987; Amici et al., 2001
**Inhibition of virus cell-to-cell transmission**	Human T-cell Leukemia Virus Type I (HTLV-1)	PGA_1_, PGJ_2_	Inhibit host cell proliferation by inducing cell arrest at the G1/S interface	D’Onofrio et al., 1992; Lacal et al., 1994a,b
**Inhibition of virus-induced inflammation**	Influenza	15d-PGJ_2_	Decrease virus-induced release of proinflammatory cytokines (IL-6, TNF-α) and chemokines (CCL2, CCL3, CCL4, and CXCL10) via PPAR-γ pathway	Cloutier et al., 2012
	Respiratory Syncytial Virus (RSV)	15d-PGJ_2_	Decrease virus-induced release of cytokines (TNF-α, GMCSF, IL-1α, IL-6), and the chemokines (CXCL8 (IL-8) and CCL5) via PPAR-γ pathway. Reduce immune cells adhesion by inhibiting virus-induced up-regulation of intercellular adhesion molecule-1 (ICAM1). Reduce activity of inflammatory pathway, NF-κB.	Arnold et al., 2007
	Human Immunodeficiency Virus-1 (HIV-1)	15d-PGJ_2_	Suppress NF-κB activation by inhibiting IKK complex	Boisvert et al., 2008
	Zika virus (ZIKV)	15d-PGJ_2_	Control brain inflammation by downregulating microglial activation and by inducing apoptosis of activated microglia	Bernardo and Minghetti, 2006

Depending on the virus, cyPGs utilize various mechanisms and act on different viral cycle events to interfere with virus production. In HIV-1 infection and avian influenza, A virus infection, cyPGs prevent very early virus infection phases such as viral adsorption and penetration into target cells (Rozera et al., 1996; Carta et al., 2014). Even though antiviral action mechanisms differ between various viruses and host cell systems, the inhibition of virus replication by cyPGs is often associated with (1) alteration in viral protein synthesis and (2) alteration in viral glycoprotein glycosylation (Table 2). PGA_1_ treatment inhibited replication of Mayaro virus (MAYV) (an arbovirus endemic to certain humid forests of tropical South America) by 95% at 24 h post-infection in human epithelial type 2 (Hep-2) cells (Caldas et al., 2018). PGA_1_ treatment inhibited viral structural protein synthesis by 15%, possibly via heat shock protein70 (HSP70) induction (Caldas et al., 2018).

### Cyclopentenone Prostaglandins Alter Viral Protein Synthesis

Inhibition of individual virus replication by cyPGs is marked by dysregulation of viral protein synthesis (Table 2). In influenza, A PR8 virus (a mouse-adapted H1N1 influenza virus causing severe infection in mice)-infected cells, treatment of Δ^12^-PGJ_2_ substantially decreased the synthesis of PR8 proteins such as hemagglutinin (HA), nucleoprotein (NP), and membrane protein M1 (Pica et al., 1993). PGA_1_ could cause a significant delay in the synthesis of late viral polypeptides: HA, membrane protein M1, structural protein M2, and non-structural protein NS2 (Conti et al., 2001). Furthermore, both studies showed that inhibition or delay of viral protein synthesis is accompanied by induction of a 70 kDa host polypeptide identified as HSP70 by immunoblot analysis (Pica et al., 1993; Conti et al., 2001). Because viral protein synthesis is repressed as long as HSP70 is present in the host cell, HSP70 seems to play an essential role in cyPGs antiviral activity.

In VSV infection, Δ^12^-PGJ_2_ can affect two distinct stages (an early stage and a late-stage) of the virus replication cycle in epithelial monkey cell lines (Pica et al., 1993). The inhibition of the virus at the initial stage is associated with altered viral protein synthesis. When the cells are treated with 8 mg/ml of Δ^12^-PGJ_2_ soon after virus infection, there is a dramatic decrease in VSV protein synthesis. Similar to the effect on influenza A virus replication, inhibition of VSV protein synthesis by Δ^12^-PGJ_2_ is also associated with the induction of a 74 kDa polypeptide belonging to the group of heat shock protein HSP70 (Pica et al., 1993). In another study, PGA_1_ treatment decreased VSV proteins’ production and the amount of respective viral mRNA (Bader and Ankel, 1990). This study found that PGA_1_ exerts its antiviral activity at the VSV genes’ primary transcription level, which leads to a reduction in viral mRNA synthesis, viral protein synthesis, and, ultimately, viral replication. To further investigate the antiviral activity of cyPGs, another study performed an RNA polymerase assay and reported that cyPGs potently inhibit VSV RNA polymerase (Parker, 1995). This inhibition correlates with the decrease in VSV replication in infected cells, indicating that cyPGs antiviral activity is due to VSV RNA polymerase inhibition.

In addition to VSV, cyPGs also exert a transcriptional block in the replication of herpes simplex virus type 1 (HSV-1) (Amici et al., 2001), HSV-2 (Yamamoto et al., 1987), and HIV-1 (Rozera et al., 1996). In HSV-1 infected human laryngeal carcinoma cells and neuroblastoma cells and HIV-1 infected colonic epithelial cells (caco-2 cells), cyPGs inhibit viral gene expression by suppressing NF-κB activation, independent of the PPAR-γ pathway (Amici et al., 2001; Boisvert et al., 2008). NF-κB is essential for many processes, including viral gene expression and, consequently, replication of viruses that contain NF-κB binding sites in their genomes. In its inactivated cytosolic form, NF-κB is bound to inhibitory IκB proteins such as IκBα. Stimuli like bacterial and viral infections increase the activity of the IKK complex, which phosphorylates IκBα, leading to ubiquitination and degradation of IκBα by proteasomes. Once NF-κB is free from IκBα, it translocates into the cell nucleus, activating the transcription of many genes, including the viral genes of HSV-1 and HIV-1 (Amici et al., 2001; Boisvert et al., 2008). Amici et al. (2001) showed that PGA_1_ significantly decreases the NF-κB induction in HSV-1 infected cells by inhibiting the IKK complex.

Similarly, another study reported that the administration of PGJ_2_ reduces IKK activity in HIV-1 infected cells (Boisvert et al., 2008). In both cases, suppression of IKK activity by cyPGs prevents IκBα degradation and NF-κB translocation to the nucleus. As a result, viral gene transcription and protein synthesis were repressed, leading to a significant reduction in virus production. In addition to interfering with NF-κB induction, cyPGs also target another pathway independent of NF-κB to inhibit HIV-1 replication. Kalantari et al. (2009) reported that 15d-PGJ_2_ represses HIV-1 transcription by inhibiting HIV-1 transactivating protein, Tat. While the host transcriptional factor NF-κB binds to the 5′ long terminal repeat (LTR) of HIV-1 to initiate transcription, viral Tat protein is recruited to an RNA stem-loop structure called transactivation response element (TAR) and is necessary for transcriptional elongation. Tat then recruits transcription elongation factor p-TEFb, which transactivates HIV LTR and allows the RNA polymerase II to continue the transcription with high processivity. 15d-PGJ_2_ interferes with Tat-dependent transcriptional elongation by covalently modifying the thiol groups of Tat’s cysteine residues (Kalantari et al., 2009). The resulting altered Tat protein is unable to transactivate HIV LTR in U937 human macrophages, inhibiting the transcription and replication of the virus.

### Cyclopentenone Prostaglandins Alter Viral Glycoprotein Glycosylation

cyPGs can also inhibit viral replication at the post-translational level by altering the glycosylation of viral glycoproteins. This is seen in the VSV and Sendai virus (Table 2). As mentioned earlier, Δ^12^-PGJ_2_ inhibits the VSV replication in the epithelial monkey cell line at two stages of the virus replication cycle. The inhibition at the early stage is due to a block in viral protein synthesis. Administration of Δ^12^-PGJ_2_ at a later stage (6–8 h post-infection) also leads to a decrease in virus production even though viral protein synthesis should have been completed by that time (Pica et al., 1993). Δ^12^-PGJ_2_ treatment started at a later stage does not affect viral protein synthesis, but it drastically decreases the glucosamine incorporation into the virus glycoprotein G without altering most cellular proteins.

Similarly, PGA_1_ treatment in AGMK cells infected with the Sendai virus results in inhibition of glycosylation of viral glycoproteins hemagglutinin-neuraminidase (HN) and fusion protein (F), as indicated by the decrease in glucosamine incorporation (Santoro et al., 1987). The synthesis of non-glycosylated viral polypeptides of RNA transcriptase complex, including proteins P, NP, and matrix protein (M), are not affected by PGA_1_ treatment. Likewise, Δ^12^-PGJ_2_ also markedly reduces the incorporation of glucosamine into HN and F viral glycoproteins without inhibiting the synthesis of cellular or viral proteins (Amici et al., 2001). The altered HN glycoprotein cannot insert into the cell membrane, which leads to an inhibition of virus maturation and production.

### The Effect of Cyclopentenone Prostaglandins on Viral Transmission

cyPGs can interfere with virus transmission via their antiproliferative activity. When PGA_1_ and PGJ_2_ are given to human T-cell leukemia virus type-I (HTLV-1) producing MT-2 cell line, they inhibit the growth of the cells in a dose-dependent manner (D’Onofrio et al., 1992). These cyPGs cause the cells to be arrested at the G1/S interface without detectable cellular toxicity. Another study showed that PGA_1_ and PGJ_2_ inhibit the proliferation of myeloid cells (K562 pluripotent stem cells, HL60 promyelocytic cells, and U937 monoblastoid cells) during early infection of HTLV-1, also in a dose-dependent manner (Lacal et al., 1994a,b). Furthermore, out of the three myeloid cell lines used in the study, the effect of growth inhibition is highest in U937 monoblastoid cells, followed by HL60 promyelocytic cells, and then K562 pluripotent stem cells. This suggests that cyPGs have a more significant antiproliferative effect on differentiated cells.

The primary mode of infection of HTLV-1 is cell-to-cell transmission (Yoshida and Seiki, 1987). Furthermore, for retrovirus-like HTLV-1, integration of proviral DNA occurs after the initiation of cellular DNA synthesis in dividing cells (Varmus et al., 1979). Thus, alterations in cell proliferation and cell cycle can affect the permissiveness of recipient cells to HTLV-1. Indeed, in U937 monoblastoid cells co-cultured with virus-donor cells, PGA_1_ and PGJ_2_ treatments reduce the transmission of HTLV-1 (Lacal et al., 1994a,b). However, in less differentiated K562 pluripotent stem cells and HL60 promyelocytic cells, infection of recipient cells increased after cyPGs treatment antiproliferative activity is observed in these cells. This suggests that the effect of cyPGs on virus transmission is affected by cell differentiation.

### The Effect of Cyclopentenone Prostaglandins on Viral Infection Induced Inflammation

Viral infections such as influenza virus, HIV-1, and respiratory syncytial virus (RSV) are characterized by excessive inflammation with the upregulation of proinflammatory cytokines and chemokines. The amount of these proinflammatory molecules correlates with the severity of illness (Griffin et al., 1994; Wesselingh et al., 1994; Hornsleth et al., 2001; Welliver et al., 2002). Given the anti-inflammatory effects of cyPGs, studies have been done to explore the possibility of utilizing cyPGs as a therapeutic agent for viral infections. In mice infected with lethal influenza infection, administration of 15d-PGJ_2_ 1 day after infection resulted in reduced influenza morbidity and mortality, accompanied by substantially decreased gene expression of proinflammatory cytokines (IL-6 and TNF-α) and chemokines (CCL2, CCL3, CCL4, and CXCL10) via activation of PPAR-γ pathway (Cloutier et al., 2012). Similarly, 15d-PGJ_2_ and other PPAR-γ agonists (ciglitazone and TGZ) can inhibit the RSV-induced release of cytokines TNF-α, GMCSF, IL-1α, IL-6, and the chemokines CXCL8 (IL-8) and CCL5 (Arnold et al., 2007). Moreover, RSV infection of the human airway epithelial cells causes an increase in expression of intercellular adhesion molecule-1 (ICAM1) on the cell surface, which enhances the adhesion of recruited immune effector cells, contributing to an intense inflammatory response and increased cytotoxicity (Wang et al., 2000; Arnold et al., 2007). Treatment of 15d-PGJ_2_ and other PPAR-γ agonists results in inhibition of the up-regulation of ICAM1, with the reduced cellular amount of ICAM1 mRNA (Arnold et al., 2007). This leads to a significant reduction in the adhesion of immune cells to RSV-infected cells. Also, the 15d-PGJ_2_ treatment in RSV-infected cells is associated with reduced activity of NF-κB, a transcription factor essential for inflammatory responses. In HIV-infected intestinal epithelial cells, 15d-PGJ_2_ also reduces the nuclear translocation of NF-κB and represses HIV-1 transcription by decreasing the activity of IKK (Boisvert et al., 2008). Overall, cyPGs can reduce the exaggerated inflammatory response associated with viral infections and great therapeutic value. PGD_2_/DP1 axis and 15d-PGJ_2_ signaling contributes to the regulation of the CNS-specific response to pathogens such as neurotropic coronavirus (CoV) (Vijay et al., 2017) and acute encephalitis (Rosenberger et al., 2004), chronic demyelinating encephalomyelitis causing neurotropic virus called “MHV” (mouse hepatitis virus strain JHM) (Zheng et al., 2020).

Zika virus (ZIKV), one of the most medically relevant viral infections, affects the developing brain during pregnancy, and its connection with congenital malformations/microcephaly is well documented (de Oliveira et al., 2019). Neuroinflammation is one of the critical factors contributing to ZIKV-related microcephaly, inflammatory processes mediated by glial cells (Wen et al., 2017; Huan et al., 2018). PGD_2_, PGE_1_, PGE_2_, and PGI_2_ have been correlated with neuroinflammation, protecting the CNS, and physiological responses to minimize further damage to neural tissue. Their anti-inflammatory reaction has been demonstrated in neuronal injuries (Shi et al., 2010) and neuroprotection during acute brain injury (Liang et al., 2005; An et al., 2014) 15d-PGJ_2_ activates PPAR-γ by downregulating microglial activation despite the proinflammatory environment because of the neural damage (Bernardo and Minghetti, 2006).

15d-PGJ_2_ has demonstrated beneficial effects in the severe diseases arising from bacterial infections of *Staphylococcus aureus* (Phulwani et al., 2006), *Salmonella enterica* Typhimurium (Buckner et al., 2013), leading to brain abscess, typhoid fever, gastroenteritis, and protozoan hemoflagellate *Trypanosoma brucei* infection-causing sleeping sickness in humans (Figarella et al., 2006).

## Other Alpha, Beta-Unsaturated Carbonyl Lipids and Cyclopentenone Isoprostanes

There is another category of highly reactive electrophilic molecules, which react and modify both proteins and DNA resulting in toxicity, protein dysfunction (Sayre et al., 2006) or tissue damage and disease progression (Lee and Park, 2013). These are α, β-unsaturated aldehydes such as acrolein (ACR), 4-hydroxy-2-non-enal (4-HNE), and crotonaldehyde (CRA) are the most reactive and toxic α, β-unsaturated aldehydes (Lee and Park, 2013). These induce toxicity because of depletion of cellular GSH and inactivation of antioxidant enzymes (GPx and thioredoxin; TRx) subsequently leading to ROS production, reactive nitrogen species (RNS), and free radicals (Stocker and Keaney, 2004; Lee and Park, 2013). Lipid peroxidation (LPO)-derived α, β-unsaturated aldehydes play an important pathophysiological role in vascular diseases by inducing the production of various atherogenic factors, inflammatory mediators, activation of NF-κB signaling pathway, redox signaling mediators leading to cellular and tissue injury (Lee and Park, 2013).

Isoprostanes (IsoPs) are PG-like compounds that are produced *in vivo* independently of COX enzymes, primarily by ROS-mediated or free radical-induced peroxidation of arachidonic acid (Stamatakis and Perez-Sala, 2006). IsoPs along with cyPGs are reactive electrophilic eicosanoids that can form covalent adducts with thiol-containing molecules, cysteine residues in proteins through Michael addition (Stamatakis and Perez-Sala, 2006). Oxidation of DHA in the central nervous system, results in the formation of IsoP-like compounds, termed neuroprostanes and are uniquely valuable to understanding the clinical pharmacology of antioxidants (Montuschi et al., 2007). Cyclopentenone IsoPs are formed abundantly in brain tissue under conditions of oxidative stress (glutathione depletion, ROS generation, activation of redox-sensitive signaling pathways) and may contribute to neuronal death causing neurodegeneration and should be addressed when designing neuroprotective therapies (Musiek et al., 2006, 2007; Porta et al., 2013). IsoPs are measured in the plasma, urine, or cerebral spinal fluid (CSF) and their increase has been observed in obese adults (Morrow, 2005; Basu, 2008), ischemia-reperfusion (Sakamoto et al., 2002; Rossi et al., 2004), Alzheimer’s disease (AD) (Montine et al., 1998, 1999a; Pratico et al., 1998, 2000), Huntington’s disease (Montine et al., 1999b), Parkinson’s disease (Fessel et al., 2003; Seet et al., 2010), and amyotrophic lateral sclerosis (ALS) (D’Amico et al., 2013). Few studies have investigated the associations between levels of F2-IsoPs and risk of breast cancer (Rossner et al., 2006), hepatocellular carcinoma (Wu et al., 2008), prostate cancer (Barocas et al., 2011; Brys et al., 2013) gastric cancer (Asombang et al., 2013). IsoPs are increased in patients with genetic disorders such as autism-spectrum disorders (Ming et al., 2005; Gorrindo et al., 2013), Smith–Lemli–Opitz Syndrome (SLOS) (Korade et al., 2013), sickle cell anemia (Akohoue et al., 2007), cystic fibrosis (Collins et al., 1999; Ciabattoni et al., 2000; Montuschi et al., 2000), Rett syndrome (RTT) (De Felice et al., 2009, 2011; Signorini et al., 2011; Durand et al., 2013), and in various inborn errors of metabolism (Mc Guire et al., 2009).

## Summary and Future Directions

There is significant evidence that cyPGs (PGA_1_, PGA_2_, and PGJ_2_), and metabolites of PGJ_2_ (15d-PGJ_2_ and Δ^12^- PGJ_2_) can induce anti-inflammatory and antiviral effects through covalent modification reactions with their α, β-unsaturated carbonyl group. cyPGs can exert anti-inflammatory and antiviral effects in various ways depending on the host cell and pathogen type. Cell type is not the only influencer on the anti-inflammatory effects of cyPGs. The concentration of cyPGs and the length/time of exposure to cyPGs have varying anti-inflammatory and antiviral effects. Based on these factors, cyPGs can show biphasic targeting of inflammation (Garzon et al., 2011). At high doses, 15d-PGJ_2_ has a dual action of stimulating anti-inflammation and anti-proliferation. Still, it can be toxic and induce both inflammation and cell proliferation at lower doses, and the biphasic pharmacodynamics has to be controlled carefully (Abbasi et al., 2016). Dose-related efficacy and safety of oral DP_2_ receptor antagonists fevipiprant (QAW039), timapriprant (OC000459), and BI 671800 have been tested in patients with allergic asthma and COPD, and PGD_2_ has shown anticancer effects in NSCLC (non-small cell lung carcinoma), kidney and lung fibrosis, and gastric cancer (Bateman Guerreros et al., 2017; Jandl and Heinemann, 2017; Pearson et al., 2017; Sandham et al., 2017a,b; Murillo et al., 2018; Brightling et al., 2020). Further research on outcomes based on specific concentrations is warranted. PPAR-γ antagonist (GW9662) and PPAR-γ ligands are new therapeutic targets in sepsis, hemorrhagic shock, and inflammation (Kaplan et al., 2005, 2010; Zingarelli and Cook, 2005; Chima et al., 2011). Synthetic PPAR-γ ligands rosiglitazone (Avandia) and pioglitazone have exhibited anti-inflammatory and antiviral effects in an EcoHIV mouse model that could decrease neurodegeneration. These drugs prove promising in treating HIV-1 associated neurocognitive disorders (Omeragic et al., 2020). This knowledge could significantly impact how viruses and inflammation can be treated.

The outcome of the 15d-PGJ_2_ treatment depends upon its exogenously administered dose as it stimulates anti-inflammation and anti-proliferation at high doses while can have toxic effects at a lower dose (Abbasi et al., 2016). Many strategies have been developed to deal with the biphasic pharmacodynamics of 15d-PGJ_2_ and one of them is using a nanoemulsion (NE) composed of triolein/distearoyl phosphatidylcholine/Tween 80 at a high encapsulation ratio (>83%) allowing slow-release kinetics (Abbasi et al., 2016). NE retained a high proportion of 15d-PGJ_2_ and directly delivered it to the cytosol, where proapoptotic targets are located, and could bypass cell membrane-associated targets involved in cell proliferation (Abbasi et al., 2016). NE could deliver 15d-PGJ_2_ to its desired site of action, excluding undesired sites, on a subcellular level (Abbasi et al., 2016) and could be used as one of the strategies for treatment. Since the use of solid lipid nanoparticles (SLN) can improve therapeutic properties by increasing drug efficiency and availability, 15d-PGJ_2_-SLN was developed and tested for its immunomodulatory potential. The 15d-PGJ_2_-SLN formulation showed good colloidal parameters, encapsulation efficiency (96%), and stability (up to 120 days) with low hemolytic effects as compared to unloaded SLN in *in vivo* experiments. The 15d-PGJ_2_-SLN formulation using low concentrations reduced neutrophil migration in three inflammation models tested. 15d-PGJ_2_-SLN increased IL-10 levels and reduced IL-1β as well as IL-17 in peritoneal fluid thus highlighting the perspectives of a potent anti-inflammatory system (de Melo et al., 2016). cyPGs have a wide spectrum of intracellular targets ranging from nuclear factors to mitochondria. Introduction of cyclopentenone moiety into molecules (jasmonates and chalcones) boosts their anticancer potential (Conti, 2006). Despite advancements made in the pharmacodynamics of cyPGs, a significant effort is needed to explore their unique therapeutic properties and tailor them to be used as leading anti-inflammatory, anticancer, and antiviral drugs.

## Author Contributions

All authors listed have made a substantial, direct and intellectual contribution to the work, and approved it for publication.

## Conflict of Interest

The authors declare that the research was conducted in the absence of any commercial or financial relationships that could be construed as a potential conflict of interest.

## Abbreviations

AD: Alzheimer’s disease
AP-1: activating protein-1
ALS: amyotrophic lateral sclerosis
AGMK: African green monkey kidney
COX: cyclooxygenase
CCR: chemokine receptors
CTL: cytotoxic T lymphocytes
CREB: cyclic AMP-responsive element-binding
cyPGs: cyclopentenone PGs
COPD: chronic obstructive pulmonary disease
DCs: dendritic cells
DGLA: dihomo g-linolenic acid
EAE: experimental allergic encephalomyelitis
EBV: Epstein–Barr virus
ERK: extracellular signal-regulated kinases
EMT: epithelial to mesenchymal transition
FRK: c-Fos -regulating kinases
GMCSF: granulocyte-macrophage colony-stimulating factor
GR: glutathione reductase
GPx: glutathione peroxidase 1
GCS: c-glutamylcysteine synthetase
HCMV: human cytomegalovirus
HDACs: histone deacetylases
HO-1: heme oxygenase-1
HSV: herpes simplex virus
Hep-2: human epithelial type 2
HSP70: heat shock protein70
HTLV-1: human T-cell leukemia virus type-I
hTERT: human telomerase reverse transcriptase
ICAM-1: intercellular adhesion molecule 1
IBD: inflammatory bowel disease
IsoPs: isoprostanes
IKK: I κ B kinase
JAK: Janus kinase
Keap1: Kelch-like ECH-associated protein 1
KSHV: Kaposi’s sarcoma herpesvirus
LBD: ligand-binding domain
mTOR: mammalian target of rapamycin
MMP-9: matrix metalloproteinase
Nrf2: NF-E2-related nuclear factor erythroid-2
NSCLC: non-small cell lung carcinoma
NE: nanoemulsion
NGS: next-generation sequencing
NLS: nuclear localization sequence
NSAIDs: non-steroidal anti-inflammatory drugs
NQO1: NAD(P)H dehydrogenase quinone 1
PAI-1: plasminogen activator inhibitor-1
PD-1: programmed cell death protein-1
PDL-1: programmed cell death ligand-1
PG: prostaglandin
PUFA: polyunsaturated fatty acid
15-PGDH: 15 − hydroxyprostaglandin dehydrogenase
PLA_2_: phospholipase A_2_
PPAR- γ: peroxisome proliferator-activated receptor-gamma
ROS: reactive oxygen species
RTT: Rett syndrome
SOCS: suppressor of cytokine signaling
SLN: solid lipid nanoparticles
SOD: superoxide dismutase
SLOS: Smith–Lemli–Opitz syndrome
TAR: transactivation response element
TGF- β: transforming growth factor- β
TGZ: troglitazone
TXA_2_: thromboxane A_2_
uPA: urokinase plasminogen activator
VEGF: vascular endothelial growth factor
VSV: vesicular stomatitis virus
VZV: varicella zoster virus.
